# Supplementary material for: WordCommentsAnalyzer: A windows software tool for qualitative research
Source: F1000Res. 2018 Sep 4;7:536. Originally published 2018 May 3. [Version 2] doi: 10.12688/f1000research.14819.2 (PMC6137411; doi:10.12688/f1000research.14819.2)
Supplement: Supplementary file 3 [file f1000research-7-17614-s0002.tgz › b2a79496-4ada-47c4-9682-a99ce867836d.zip!/Eight Iranian Health Journals/Archives of Rehabilitation_53.docx]

© Archives of Rehabilitation. This work is licensed under CC BY-NC 4.0 (https://creativecommons.org/licenses/by-nc/4.0/).

Volume & Issue: دوره 7 - شماره 3

Title: آشنایی با فرایند توان‌بخشی بینایی

http://rehabilitationj.uswr.ac.ir/article-1-40-fa.html

با توجه به تعداد کم بینایانی که همه روزه درکلینیکهای چشم پزشکی مورد معاینه قرار می‌گیرند و از طرفی عدم وجود درمـان قـطعی برای افزایـش بینـایی ایشان، ضرورت توجه به تـوانبخشی بـینایی این گـروه احساس می‌شود. در این مقاله سعی شده ابتدا به مفهوم و سپس به مراحل توانبخشی بینایی که در مراکزی با همین نام قابل انجام است پرداخته شود. در اینجا پس از ارائه آمار موجود در مورد کم بینایان و نابینایان، به چگونگی جمع آوری اطلاعات اشاره شده و پس از ارائه تعاریف استاندارد، به توضیح تیم توانبخشی پرداخته شده است . علل کم بینایی و بیماریهای چشمی که منجر به این حالت می‌شود اعم از مادرزادی، ژنتیک و اکتسابی و همچنین علل اصلی مورد توجه قرار گرفته اند. تکنیک‌های انجام معاینات و وسایلی که به عنوان وسایل کمک بینایی معروف هستند نیز از نکاتی است که در این مقاله به آن پرداخته شده است. در طی فرایند توانبخشی بینایی پس از آنکه درمانگران (اعم از متخصصین چشم و اپتومتریستها) از طریق درمانهای رایج همچون جراحی، دارو درمانی و تجویز عینکهای معمولی، موفق به افزایش بینایی بیمار و یا درمان وی نمی‌شوند، باید برای بررسی امکان افزایش دید، بیمار را به متخصصین مربوط به توانبخشی بینایی ارجاع دهند. این افراد همان اپتومتریستها و یا چشم پزشکانی است که دوره های مربوطه را گذرانده اند. در توانبخشی بینایی پس از ارزیابی میزان دید باقی مانده، اقدام به تجویز وسایل کمک بینایی مناسب می‌شود. همچنین مشاوره لازم در خصوص شغل مناسب، تکنیکهای آموزشی بخصوص در کودکان سنین مدرسه، به عمل می آید. در صورتی که دید بیمار آنقدر کم باشد که وسایل کمک بینایی مناسبی برای وی وجود نداشته باشد، بیمار به مراکز توانبخشی نابینایان ارجاع می گردد. آنچه که قابل تأمل است، خلاء مراکز توانبخشی بینایی بین سیستم درمانی و سیستم توانبخشی نابینایان است.‌

Title: شناخت مقبولیت روش‌های موجود پیشگیری از اعتیاد و ارائه الگوی مطلوب

http://rehabilitationj.uswr.ac.ir/article-1-39-fa.html

هدف: تحقیق حاضر شیوه‌های موجود پیشگیری از اعتیاد را مورد مطالعه قرار داده و نگرش افراد مختلف را نسبت به روش‌های پیشگیری بررسی کرده است. روش بررسی: در این تحقیق از روش توصیفی و پیمایشی (survey) استفاده شد، یعنی با استفاده از پرسش‌نامه نسبت به سنجش آگاهی و نگرش افراد مورد مطالعه در مورد روش‌های پیشگیری پیشنهادی و موجود و مقبولیت آن‌ها اقدام گردید. برای طراحی پرسش‌نامه ابتدا با متخصصان و صاحب نظران این حوزه‌ مصاحبه‌های باز صورت گرفت و با توجه به نظرات آنان پرسش‌نامه نهایی با 61 سئوال بسته و 2 سئوال باز طراحی شد. نمونه‌های تحقیق 2500 نفر از افراد 13 تا 35 ساله کل کشور می‌باشند که با نمونه‌گیری تصادفی از 15 استان که استانها هم تصادفی انتخاب شده بودند گزینش شدند. یافته‌ها: نتایج تحقیق نشان داد که نگرش کلی افراد نسبت به روش‌های پیشگیری مطرح شده در پژوهش و مقبولیت آن‌ها مثبت است، هر چند بین میزان مقبولیت روش‌ها تفاوت‌هایی وجود دارد که بنا بر نظر آزمودنی‌ها روش‌های رشد و توسعه فرهنگی، آموزش مهارت‌های زندگی و ارائه‌ مشاوره به مردم از مقبولیت بیش‌تری برخوردارند. نتیجه‌گیری: آزمودنی‌ها مقبولیت روش‌های موجود و پیشنهادی پیشگیری از اعتیاد را تعاملی می‌دانند که بر یکدیگر تأثیرات مستقیم و غیرمستقیم دارند که با توجه به این یافته‌ها الگوی مطلوب الگویی تلفیقی است.

Title: بررسی تأثیر تمرین‌ ذهنی بر هماهنگی چشم و دست در دختران سالم 25-20 سال

http://rehabilitationj.uswr.ac.ir/article-1-38-fa.html

هدف: انسانها دارای توانایی ایجاد ارتباطات ذهنی بین فرآیندهای درکی و حرکتی می‌باشند، بدون اینکه محرک خارجی این فرآیندها را تحریک کرده باشد. این توانایی را تصور حرکت می‌گویند. تمرین ذهنی کسب یک مهارت فیزیکی از طریق تصور حرکت است. به عبارت دیگر یک نوع تمرین شناختی در غیاب عملکرد حرکتی فیزیکی واقعی است . پژوهش حاضر با هدف بررسی تأثیر تمرین ذهنی بر هماهنگی چشم و دست در دختران سالم 25-20 سال صورت گرفته است. روش بررسی: این پژوهش به صورت یک مطالعه شبه تجربی آینده‌نگر کاربردی صورت گرفته است. جامعه پژوهش 45 نفر از دختران سالم 25- 20 سال دانشگاه علوم پزشکی اهواز بوده‌اند که بصورت تصادفی انتخاب شدند. ابزار پژوهش دستگاه E-Link آزمایشگاه تعقیب کنائی حرکت دانشکده توانبخشی اهواز بود. در این پژوهش هماهنگی چشم و دست بعد از تمرین فیزیکی و تمرین ذهنی مورد بررسی قرار گرفت. افراد به طور تصادفی به سه گروه تقسیم شدند: گروه فیزیکی، که افراد به مدت 10 جلسه تمرین را به صورت فیزیکی انجام می‌دادند. گروه تمرینی ذهنی، که به صورت ذهنی مراحل انجام تمرین را تصور می‌کردند و گروه کنترل، که هیچ‌گونه تمرین را انجام نمی‌دادند. یافته‌ها: نتایج به دست آمده نشان داد که گروه تمرین فیزیکی بیشترین پیشرفت را در هماهنگی چشم و دست پیدا کرده بود (P<0/05). در گروه تمرین ذهنی هم پیشرفت معنی‌داری در هماهنگی چشم و دست دیده می‌شد اما این پیشرفت کمتر از میزان پیشرفت گروه فیزیکی بود (P>0/05) گروه کنترل، پیشرفت آماری معنی‌داری رانشان نداد (P<0/05). نتیجه‌گیری: یافته‌های پژوهش این نظریه را تأیید می‌کند که تمرین ذهنی باعث تسهیل یادگیری حرکتی می‌شود. لذا توصیه می‌شود هنگامی که تمرین فیزیکی به هر دلیلی برای افراد قابل انجام نباشد از تمرین ذهنی استفاده شود.

Title: مقایسه کنش‌ها و توانش‌های کاربردی زبان در دانش‌آموزان کم‌شنوای شدید با دانش‌آموزان شنوا

http://rehabilitationj.uswr.ac.ir/article-1-37-fa.html

هدف: با توجه به اینکه دانش‌آموزان کم‌شنوا از کنش‌ها و توانش‌های کاربردی کمتری برخوردارند، لذا پژوهش حاضر به مقایسه این جنبه‌های کاربردشناسی زبان در دانش‌آموزان مدارس باغچه‌بان با دانش‌آموزان شنوای مدارس شهر تهران می‌پردازد. روش بررسی: تعداد 70 دانش‌آموز کم‌شنوای دختر و پسر با افت شنوایی شدید (90 - 70 دسی‌بل) با میانگین معدل تحصیلی 18 - 15 در پایه‌های تحصیلی اول دبیرستان تا پیش‌دانشگاهی با نمونه‌گیری هدفمند انتخاب شدند. سپس این دانش‌آموزان، با 70 دانش‌آموز شنوای همگن از نظر جنس، پایه تحصیلی، نوع رشته تحصیلی و محله زندگی که بر اساس جورکردن انتخاب شده بودند مقایسه شدند. روش پژوهش مقایسه‌ای از نوع مقطعی بوده است. ابزار پژوهش عبارتند از : پرسشنامه و آزمون کنش‌هاو توانش‌های کاربردی (شامل: درک و بیان استعاره - درک و بیان ضرب‌المثل - درک کنش گفتارهای غیرمستقیم - درک استنباط معنی - انتخاب بهترین عنوان و درک جملات طنزآمیز). جهت تجزیه و تحلیل داده‌ها از آزمون‌های کروسکال والیس، من ویتنی یو، آزمون تی و تی زوجی استفاده شد. یافته‌ها: یافته‌های پژوهش نشان داد که بین میانگین نمره کل کنش‌ها و توانش‌های کاربردی زبان در دو گروه کم‌شنوا و شنوا تفاوت معنی‌داری وجود دارد (P<0/05). همچنین بین میانگین نمره کل کنش‌ها و توانش‌های کاربردی زبان در گروه کم‌شنوا بین پایه‌های تحصیلی اول دبیرستان تا پیش‌دانشگاهی تفاوت معنی‌داری وجود دارد (P<0/05). در صورتی که بین میانگین نمره کل کنش‌ها و توانش‌های کاربردی زبان در گروه شنوا بین پایه‌های تحصیلی اول دبیرستان تا پیش‌دانشگاهی تفاوت معنی‌دار نیست (P<0/05). نتیجه‌گیری: دانش‌آموزان کم‌شنوا نسبت به دانش‌آموزان شنوا توانش‌های کاربردی زبان را کمتر درک می‌کنند. همچنین در بافتها و موقعیت‌های مختلف قادر به استفاده مناسب از آن‌ها نیستند.

Title: غربالگری جهش‌های ژن 2GJB در ناشنوایان غیرسندرمی جسمی مغلوب در استان خوزستان

http://rehabilitationj.uswr.ac.ir/article-1-36-fa.html

هدف: کاهش شنوایی 1 نفر از هر 1000 تا 2000 کودک تازه متولد شده را تحت تأثیر قرار می‌دهد. بیش از 50% از این موارد را به عوامل ژنتیکی نسبت می‌دهند. کاهش شنوایی غیر سندرمی بیش از 70 درصد از موارد ناشنوایی ارثی را شامل می‌شود که 85 درصد از آن را وراثت جسمی مغلوب تشکیل می‌دهد و تا کنون بیش از یکصد جایگاه (locus) برای این نوع ناشنوایی برآورد شده است. ژن‌های مختلفی با این ناشنوایی در ارتباط هستند که عمده‌ترین آنها جهش در ژن کانکسین 26 (CX26) می باشد که در جمعیت های مختلف وجود دارد. جهش در این ژن سبب کاهش شنوایی غیر سندرمی اتوزومی مغلوب می‌شود. هدف این مطالعه غربالگری بیماران برای جهش‌های ژن کانکسین 26 با استفاده از تکنیک PCR / ARMS بود. روش بررسی: در این مطالعه تحلیلی از افراد ناشنوا یا کم شنوای مراجعه کننده به مراکز بهزیستی شهرستانهای استان خوزستان به منظور مشاوره ژنتیک، 50 نفر بطور تصادفی انتخاب و 10-5 میلی لیتر خون از آنها گرفته شد و پس از استخراج DNA کیفیت و کمیت آن با استفاده از اسپکتروفتومتری مورد بررسی قرار گرفت. جهش del G 35 با روش ARMS/PCR مشخص و نمونه‌های هموزیگوت آن کنار گذاشته شد. نمونه‌هایی که هتروزیگوت یا منفی بودند با روش DHPLC و Direct Sequencing بررسی شدند. یافته‌ها: در این تحقیق 100 کروموزوم (50 فرد بیمار) بررسی شد: 13 کروموزوم (%13) در ژن 2GJB جهش داشتند که بالاترین شیوع به جهش del G 35 مربوط می شد. سایر جهش‌ها که در این منطقه دیده شد شامل: H127R ، (AT) 2del300، H32R و P184R بودند. همچنین پلی مورفیسم I153V در 3 کروموزوم شناسایی گردید و پلی مورفیسم V52V نیز در یک خانواده مشاهده شد. نتیجه‌گیری: این آمار با آمار سایر کشورهای جهان مشابهت چندانی ندارد و بیانگر وجود احتمالی ژن‌ها و لوکوس های دیگر دخیل در ایجاد ناشنوایی غیر سندرمی اتوزومی مغلوب در این منطقه می‌باشد.

Title: آموزش در خانواده: راهبرد اصلی توان‌بخشی مبتنی‌بر جامعه در ایران

http://rehabilitationj.uswr.ac.ir/article-1-34-fa.html

هدف: هدف این مطالعه بررسی اثر بخشی برنامه آموزش در خانواده از طرح توانبخشی مبتنی بر جامعه بر معلولین تحت پوشش شبکه های بهداشتی درمانی 21 شهرستان پایلوت که تحت آموزش قرار گرفته و پس از اتمام دوره ارزشیابی شده‌اند می‌باشد. روش بررسی: در یک مطالعه مقطعی614 معلولی که از زمان آغاز طرح، برنامه «آموزش در خانواده» را دریافت کرده بودند و از نظر متغیر وضعیت پیشرفت توانائیها، توسط کارشناسان طرح توانبخشی مبتنی بر جامعه ارزشیابی شده بودند به صورت نمونه گیری تصادفی طبقه بندی شده انتخاب شدند . داده های متغیرهای سن، جنس، نوع معلولیت، وضعیت اشتغال و نسبت آموزش دهنده به آموزش گیرنده از پرونده های این معلولین استخراج گردید. آنالیز آماری با استفاده از آزمون کای‌اسکوئر انجام گرفت. یافته‌ها: بین وضعیت پیشرفت توانائیهای افراد تحت پوشش با گروه سنی و گروه معلولیت رابطه معنادار مشاهده شد (P=0/014 و P<0/001). بهترین نتایج در گروههای سنی پائین‌تر، معلولین گروه بینایی، بیماران مصروع و معلولین گروه یادگیری و کمترین نتایج در گروههای سنی افراد کهنسال، معلولین گروه ترکیبی و معلولین شنوایی و گویایی مشاهده شد. بین وضعیت پیشرفت یا عدم پیشرفت توانائیهای افراد تحت پژوهش با نسبت آموزش‌دهنده رابطه مشاهده شد (P=0/038). معلولینی که خود مربی خود باشند بیشترین میزان پیشرفت و مربیانی که از بستگان درجه اول، دوم ویا گروه بهورزان نباشند کمترین میزان پیشرفت را دارا بودند . نتیجه‌گیری: «آموزش در خانواده» در برنامه توانبخشی مبتنی بر جامعه روشی مؤثر جهت ارتقاء عملکرد معلولین در برخی گروههای انتخابی است .

Title: بررسی تأثیر اجرای توان‌بخشی قلبی در منزل بر کیفیت زندگی بیماران مبتلا به سکته قلبی

http://rehabilitationj.uswr.ac.ir/article-1-33-fa.html

هدف: پژوهش حاضر با هدف تعیین تأثیر اجرای توانبخشی قلبی در منزل بر کیفیت زندگی بیماران مبتلا به سکته قلبی انجام گرفت. روش بررسی: در این پژوهش نیمه تجربی 38 بیمار مبتلا به سکته قلبی بدون عارضه براساس شرایط لزوم ورود به مطالعه انتخاب شده و بصورت تصادفی به دو گروه مداخله (18 نفر) و کنترل (20 نفر) تقسیم شدند. اطلاعات با استفاده از پرسشنامه سنجش آگاهی بیمار، کیفیت زندگی مک نیو و چک لیست کنترل و پیگیری بیمار در منزل با روش مصاحبه و مشاهده جمع‌آوری گردید.گروه مداخله تحت آموزش نظری و عملی «برنامه توانبخشی قلبی در منزل» قرار گرفت و در انتها نیز کتابچه آموزشی ارائه شد. تداوم اجرای عملی برنامه توسط واحدهای پژوهش، حین سه ملاقات در منزل به فاصله یک، دو و سه ماه پیگیری گردید و مشاوره و راهنمایی و پیگیری تلفنی نیز انجام شد. برای بیماران گروه کنترل، کتابچه آموزشی در انتهای پژوهش ارائه گردید. یافته‌ها: یافته‌های پژوهش نشان دهنده افزایش کیفیت زندگی بیماران گروه مداخله پس از انجام توانبخشی قلبی در منزل در ابعاد جسمی، روحی - روانی و کل بوده است (P=0/000). در بعد اجتماعی افزایش معنی‌داری بعد از مداخله مشاهده نگردید (P=0/308). در گروه کنترل کاهش در میانگین‌ها در ابعاد مختلف کیفیت زندگی مشهود بود لیکن این کاهش تنها در بعد اجتماعی معنی‌دار بوده است (P=0/009). در مقایسه دو گروه، قبل از مداخله در هیچ یک از ابعاد کیفیت زندگی تفاوت معنی‌داری مشاهده نشد. مقایسه کیفیت زندگی دو گروه بعد از مداخله نیز فقط در بعد روحی- روانی دارای تفاوت معنی‌دار آماری بوده است (P=0/032). نتیجه‌گیری: با توجه به عدم تفاوت معنی‌دار میانگین کیفیت زندگی در گروه مداخله و کنترل قبل از مداخله، می‌توان تفاوت میانگین قبل و بعد کیفیت زندگی در ابعاد جسمی، روحی- روانی و کل در گروه مداخله را به اثر بخشی مداخله نسبت داد، لیکن فرضیه تحقیق مبنی بر تأثیر توانبخشی قلبی در منزل بر کیفیت زندگی بیماران مبتلا به سکته قلبی حمایت نشد. این نتیجه را می‌توان به ماهیت پویا، ذهنی و چند بعدی مفهوم کیفیت زندگی و نیاز به نمونه بزرگتر مطالعه و زمان طولانی‌تر برای پیگیری بیماران، نسبت داد.

Title: بررسی عوامل خطرساز در ابتلا به اختلال بیش‌فعالی‌/کمبود توجه

http://rehabilitationj.uswr.ac.ir/article-1-32-fa.html

هدف: با توجه به اهمیت بالینی اختلال بیش‌فعالی/ کمبود توجه به‌عنوان یکی از شایع‌ترین اختلالات رفتاری در کودکان و تأثیر آن در سیستم خانواده در پژوهش حاضر به بررسی عوامل خطرساز در ابتلا به این اختلال پرداخته می‌شود. روش بررسی: پژوهش حاضر از نوع تحلیلی مقایسه‌ای بوده و آزمودنی‌های پژوهش حاضر را 192 کودک مبتلا به اختلال بیش‌فعالی/ کمبود توجه که با استفاده از روش نمونه گیری در دسترس در یک دوره زمانی دو ساله(ابتدای سال 82 تا انتهای سال 83) به مرکز روانپزشکی اطفال ارجاع شده بودند و تشخیص اختلال بیش‌فعالی/ کمبود توجه بر اساس معیارهای DSM-IV توسط دو روانپزشک کودک در آنها مسجل شده بود (گروه آزمایش) و 210 کودک جامعه عادی که با استفاده از روش نمونه گیری تصادفی از مدارس و مهدهای کودک شهر تهران انتخاب شده و از لحاظ متغیرهای پژوهش با گروه آزمایش همتاسازی شده بودند (گروه کنترل)، تشکیل می‌دادند. داده‌های پژوهش با کمک روش‌های توصیفی مانند فراوانی و درصد و نیز استفاده از روش‌های آمار استنباطی نظیر خی‌دو مورد تحلیل قرار گرفتند.   یافته ها: یافته‌ها نشان داد جنسیت فرزندان یک عامل مهم در بروز این اختلال می باشد و میزان بروز این اختلال در پسران از دختران بیشتر است (P=0/0001 و X2=33/8). خویشاوندی والدین یک عامل خطرساز در بروز این اختلال می باشد و میزان بروز ADHD در فرزندان والدین دارای نسبت خویشاوندی از فرزندانی که والدین غیرخویشاوند دارند بیشتر است (P=0/03 و X2=4/95). میزان بروز اختلال ADHD در کودکانی که سابقه ابتلا به اختلال بیش فعالی در والدین و بستگان آنها وجود دارد بیشتر از کودکانی است که سابقه ابتلا به اختلال بیش فعالی در والدین و بستگان آنها وجود ندارد (P=0/0001 و X2=26/88). دارا بودن سابقه بیماری‌های روانی در والدین نیز یک عامل خطرساز در بروز این اختلال می باشد و میزان بروز اختلال ADHD در کودکان والدینی که دارای سابقه بیماری های روانی می باشند بسیار بیشتر از کودکان والدینی است که سابقه بیماری های روانی ندارند (P=0/0001 و X2=9/83). در نهایت میزان بروز اختلال ADHD در فرزندان اول و دوم بسیار بیشتر از فرزندان سوم و بالاتر است (P=0/0001 و X2=23/41). نتیجه‌گیری: پسر بودن، ازدواج خویشاوندی، سابقه ابتلا به اختلال بیش فعالی در والدین و بستگان، سابقه بیماری های روانی در والدین و فرزند اول و دوم بودن از عوامل خطرساز مهم در بروز ADHD می باشد.

Title: تجربیات مادران از داشتن کودک عقب‌مانده ذهنی

http://rehabilitationj.uswr.ac.ir/article-1-35-fa.html

هدف: عقب ماندگی ذهنی علاوه بر شیوع قابل توجه، با نقصان رشد در ابعاد مختلف جسمانی، روانی، تکاملی، اجتماعی و تربیتی همراه بوده و می‌تواند تأثیرات نامطلوبی بر ساختار و عملکرد خانواده داشته و باعث تجربه این تنش‌ها به صور مختلف توسط پدر و مادر شود. از آنجا که بررسی‌ها و نتایج حاصل از آنها می‌تواند در شناخت این مشکلات و حتی نحوه چگونگی اداره آنها کمک مؤثری باشد. لذا، این تحقیق کیفی به توصیف ساختار تجربیات مادران این کودکان، و به معانی که آنها از داشتن یک کودک عقب‌مانده ذهنی دارند پرداخته است. روش بررسی: برای توصیف تجربه مادران از تحقیق کیفی به روش پدیده شناسی برای جمع‌آوری داده‌ها و آنالیز آن استفاده شد. در این مطالعه با نمونه‌گیری هدفمند 12 مصاحبه باز با مادران دارای یک کودک عقب‌مانده ذهنی آموزش پذیر شاغل به تحصیل در مدرسه استثنایی آموزش و پرورش به عمل آمد. مصاحبه‌ها ضبط شد و عیناً بصورت دست‌نوشته‌هایی درآمد و با روش کولازی آنالیز شد. یافته‌ها: از میان 336 جمله با معنی (مضمونی) 6 مضمون اصلی بدست آمد که عبارتند از: برخورد با ناتوانی تکاملی کودک، احساسات منفی، کودک محور سلامتی و ناسلامتی مادر، عدم توانایی در مدیریت خود و کودک (مستأصل شدن مادر)، حمایت همسر، خانواده و دیگران، مبهم بودن آینده (قدم در تاریکی برداشتن). نتیجه‌گیری: تجربیات مادران ازداشتن کودک عقب‌مانده ذهنی نشان می‌دهد که آنان کودکانشان را نعمت و امانت الهی می‌دانند و برای پذیرش و سازگاری با وجود او تنها به خدا توکل دارند و از منابع حمایتی مناسب و اثربخشی برخوردار نیستند. بعلت تقبل مسئولیت بیش از اندازه در طول زندگی از جان مایه می‌گذارند که نتیجه آن تحمل استرس زیاد، احساس ناامیدی، غم و اندوه و درک ابهام آمیز از آینده کودک و خود است. ایفای نقش اول در حمایت همه جانبه کودک موجب غفلت آنان از خود در همه ابعادجسمی، روحی، روانی و اجتماعی شده است.
